# Multi-ancestry population attributable risk assessment of common genetic variation in Alzheimer’s and Parkinson’s diseases

**DOI:** 10.1101/2024.09.23.24314240

**Published:** 2024-09-25

**Authors:** Lietsel Jones, Catalina Cerquera-Cleves, Artur FS Schuh, Mary B Makarious, Hirotaka Iwaki, Mike A. Nalls, Alastair J Noyce, Global Parkinson’s Genetics Program (GP2), Cornelis Blauwendraat, Andrew Singleton, Ignacio Mata, Sara Bandres-Ciga

**Affiliations:** 1. Center for Alzheimer’s and Related Dementias (CARD), National Institute on Aging and National Institute of Neurological Disorders and Stroke, National Institutes of Health, Bethesda, MD, USA.; 2. DataTecnica LLC, Washington DC, USA.; 3. Department of Neurosciences, Neurology Unit, Hospital Universitario San Ignacio, Bogotá, Colombia; 4. CHU de Québec Research Center, Axe Neurosciences, Université Laval, Quebec City, Canada; 5. Departamento de Farmacologia, Universidade Federal do Rio Grande do Sul, Porto Alegre, Brazil; 6. Serviço de Neurologia, Hospital de Clínicas de Porto Alegre, Porto Alegre, Brazil; 7. Centre for Preventive Neurology, Wolfson Institute of Population Health, Queen Mary University of London, London, UK.; 8. Laboratory of Neurogenetics, National Institute on Aging, Bethesda, Maryland, USA; 9. Genomic Medicine, Lerner Research Institute, Cleveland Clinic Foundation, Cleveland, OH, United States.

**Keywords:** genetic risk, population attributable risk, genome-wide association, diverse ancestries, clinical trials, target prioritization, Alzheimer’s disease, Parkinson’s disease

## Abstract

Multiple scientific studies, mostly performed within European populations, have unraveled many of the genetic factors associated with Alzheimer’s disease (AD) and Parkinson’s disease (PD) etiologies, improving our understanding of the molecular pathways implicated in the pathogenesis of these conditions. However, there is increasing evidence that the genetic architecture of these diseases differs across ancestral populations. This raises concerns about the efficacy of therapeutic interventions crafted around genetic targets prevalent only in European ancestry populations. Such interventions neglect potentially distinctive etiological profiles, including Latino, Black/African American, and East Asian populations. In the current study, we explore Population Attributable Risk (PAR) in AD and PD etiologies and assess the proportion of disease attributed to specific genetic factors across diverse populations. Leveraging data from genome-wide association studies across four ancestries, we explore distinct and universal therapeutic targets across diverse populations. Multi-ancestral genetics research is critical to the development of successful therapeutics and treatments for neurodegenerative diseases. By offering insights into genetic disparities, we aim to inform more inclusive and effective therapeutic strategies, advancing personalized healthcare.

## Introduction

Alzheimer’s disease (AD) and Parkinson’s disease (PD) are the most prevalent neurodegenerative disorders globally; their incidence is expected to triple by 2050, particularly in low to middle-income countries ^1,2^. Genome-wide association studies (GWAS) have uncovered key genomic regions associated with the risk and pathogenesis of these conditions across diverse ancestries ^3–10^. These advances are crucial for future therapeutic interventions based on genetic targets, considering that drug mechanisms with genetic support have a 2.6x higher probability of success than those without^11^. Genetic evidence has proven particularly valuable in areas where existing treatments fail to modify disease progression, providing a solid foundation for developing new transformative drugs^11^. However, the majority of GWAS on neurodegenerative diseases predominantly feature participants of European ancestry, underrepresenting the ancestral diversity of non-European populations^12^. There is an urgent need for improved drug development target selection and prioritization methods that consider the different genetic architecture across all ancestries.

Population Attributable Risk (PAR) is a statistical measure used in epidemiology to estimate the proportion of disease cases that would decrease if a risk factor were removed from a population ^13^. PAR estimates how much disease occurrence is influenced by particular risk factors, offering insights into disease pathophysiology and therapeutic strategies to mitigate these risks. In genetics, PAR represents a comparative means for comparing risk across genetic loci relative to the odds ratio and frequency of the most representative variant(s) in a given region. PAR serves to prioritize genetic targets according to their impact on a specific population^14^. By understanding PAR in the genetic context of AD and PD, healthcare strategies and policies can be tailored to the specific needs of diverse population groups.

In this study, we aimed to comprehensively explore PAR estimates for common genetic risk factors associated with AD and PD through GWAS across various populations. Our goal was to uncover disparities in genetic predispositions and their implications for precision therapeutic development and applicability across genetic ancestry groups in order to investigate distinct and universal therapeutic targets. Identifying these disparities may help the field to develop more equitable and effective therapies, ensuring that therapeutic interventions are tailored to the genetic makeup of diverse populations.

## Methods

### Data sources

Our reference datasets consisted of summary statistics from previously published population-specific GWAS conducted in AD and PD across diverse genetically defined ancestries ^3–10^. A detailed summary of these data can be found in Table S1. To prevent the possible weakening of associations or biased analyses, we did not include summary statistics using proxy cases (those from biobanks without a clinically defined case status). The variants in this study are independent risk alleles and did not include those in linkage disequilibrium. Information about data access and all scripts for analyses are publicly available on GitHub (DOI 10.5281/zenodo.13774455; https://github.com/GP2code/PAR-ADPD/).

### Population Attributable Risk calculations

In this context, PAR is a measure of the risk reduction achieved by hypothetically removing a risk allele from the risk-associated locus^14^. It is a combination of the magnitude of risk association and the frequency of the risk allele. Before conducting PAR calculations, we filtered each dataset by p < 0.05 for nominally significantly associated variants. This refined filter enabled us to enhance confidence in the directionality of effect estimates for risk alleles and minimize potential sources of bias. We filtered summary statistics based on overlap with the most significant disease variants per locus from recent multi-ancestry GWAS models^8^. Odds ratios (ORs) were computed from beta estimates for each ancestry group contributing to the GWAS meta-analysis. We standardized beta estimates to refer to the same effect allele across different ancestries. These odds ratios were essential for assessing the strength and direction of the association between single-nucleotide polymorphisms (SNPs) and disease status. The risk allele frequency (RAF) was calculated across ancestries for SNPs representing significant loci. This frequency was utilized in further calculations of the PAR for each locus in the respective populations.

We calculated PAR using the formula:

$$PAR=\frac{p(OR-1)}{p(OR-1)+1}$$

Where p represents the RAF and OR is the calculated odds ratio for each risk allele (OR > 1, beta > 0), both extracted from summary statistics under study. RAF values were plotted against their corresponding ORs for each ancestry group.

## Results

A total of 55 AD and 90 PD variants from GWAS meta-analyses^3–10^ were evaluated as population-attributable risk factors for European, Black/African American, Latino, and East Asian ancestries (Table S2, Table S3). Table S4 highlights the variants with the highest PAR estimates for both AD and PD in each population.

### Cross-ancestry population attributable risk comparison reveals the universally applicable genetic targets for Alzheimer’s disease and Parkinson’s disease

**Figure 1.**
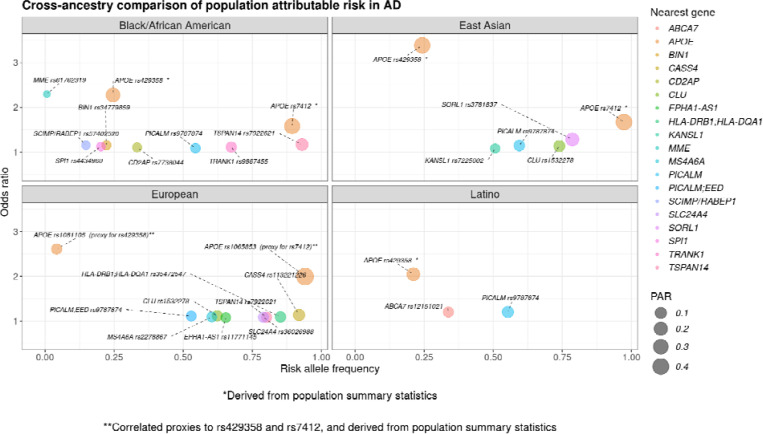
Highest population attributable risk (PAR) for Alzheimer’s disease-related variants in Black/African American, East Asian, European, and Latino populations, respectively. The x-axis represents the risk allele frequency, and the y-axis represents the odds ratio, or effect size. The nearest gene for each variant is indicated by color with the size of each dot being corresponding to the PAR value.

For AD, two loci had the highest PAR across multiple ancestries. The *TSPAN14* locus represented by the rs7922621 variant had one of the top signals in both European and Black/African American ancestries. The rs9787874 variant at the *PICALM* locus also had one of the top PAR estimates in individuals of Latino, East Asian, Black/African American, and European ancestry (Figure 1)(Table S4).

**Figure 2.**
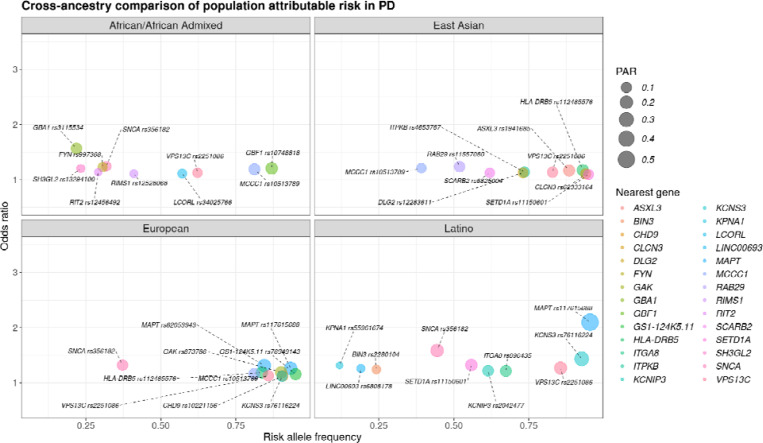
Highest population attributable risk (PAR) for Parkinson’s disease-related variants in African/African Admixed, East Asian, European, and Latino populations, respectively. The x-axis represents the risk allele frequency, and the y-axis represents the odds ratio, or effect size. The nearest gene for each variant is indicated by color with the size of each dot being corresponding to the PAR value.

In relation to PD, different variants exhibited nominal significance in cross-ancestry PAR estimates. *SNCA* locus represented by the rs356182 variant was identified among top PAR signals for European, African/Admixed, and Latino populations. Top hits in the *MAPT* locus were identified among Latinos and Europeans, while higher PAR estimates in the *SNCA* locus were found among individuals of Latino, European, African/African Admixed, and East Asian descent. Moreover, the rs10513789 variant at the *MCCC1* locus was found among the top signals in the African/African Admixed, European, and East Asian ancestry populations; and *VPS13C* rs2251086 was among the highest PAR estimates in all populations (Figure 2)(Table S4).

### APOE, GBA1, and LRRK2 display varying population-attributable risks for AD and PD across diverse ancestries

**Figure 3.**
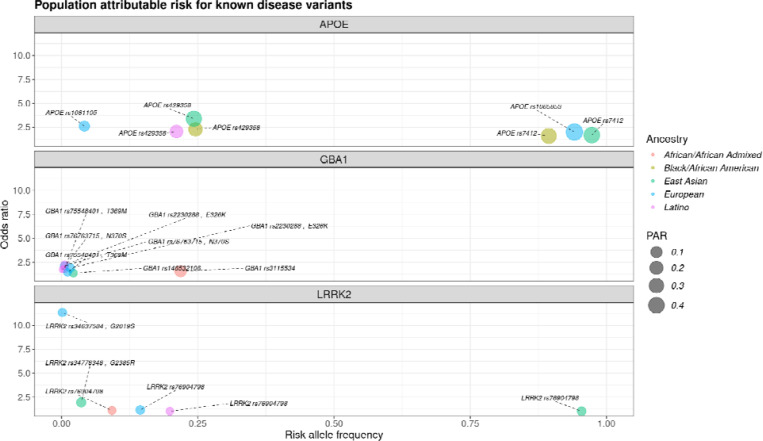
Population attributable risk (PAR) comparison among assessed populations for known disease variants: APOE, GBA1, and LRRK2, respectively. The x-axis represents the risk allele frequency, and the y-axis represents the odds ratio, or effect size.

The *APOE* allelic variants (rs7412 and rs429358), widely recognized as the major genetic risk factors for AD, consistently demonstrated higher PAR estimates across all ancestries than other genetic loci (Figure 3). Notably, individuals of Black/African American and East Asian descent exhibited the highest PAR values for both variants compared to other ancestries, whereas those of European ancestry showed lower values for the rs1081105 variant (a proxy for rs429358) than other ancestries. In addition, individuals of Latino ancestry displayed the lowest PAR for *APOE* compared to other ancestries, with rs429358 emerging as the highest *APOE* variant in this group, and rs7412 not being present in the summary statistics under study (Table S4).

On the other hand, *GBA1* was identified as one of the top PAR signals in the African/African Admixed ancestry, primarily driven by the population-specific variant *GBA1* rs3115534. In contrast, other coding variants demonstrated varying PAR estimates across ancestries (Table S5). PAR estimates for *GBA1* rs76763715 tagging p.N370S were lower in Europeans and Latinos, as well as the estimates for the intronic *GBA1* rs146532106 variant in the East Asian population (Figure 3)(Table S3). Low PAR estimates were observed for the *LRRK2* variant rs76904798 in all ancestries assessed.

## Discussion

This study aimed to dissect the impact of cross-ancestral risk alleles for AD and PD at a population scale by assessing and comparing PAR for variants from GWAS meta-analyses across diverse ancestries, including European, African, East Asian, and Latino. While PAR fractions have previously been studied for modifiable risk factors in neurodegenerative diseases^15,16^, to the best of our knowledge, this is the first study to estimate the PAR for AD/PD-related genetic variation across ancestries. By leveraging summary statistics from multi-ancestry GWAS, we assessed the genetic variants with the greatest influence on risk of AD/PD based on PAR estimations in a global setting.

In AD, East Asian and European ancestry populations exhibited the highest PAR estimates for *APOE* compared to other populations, underscoring the well-established risk associated with *APOE4* in these groups^8^. In contrast, *APOE* risk varies considerably among Latino and Black/African American populations. Recent studies have provided insights into the differential risk associated with *APOE4* risk alleles among populations of African ancestry. African-descent populations carrying the *APOE4* allele have been found to have a lower AD risk than other populations with this same allele, suggesting that the African ancestral genetic background surrounding the *APOE* gene is associated with a lower odds ratio for risk variants^17^. A recent study identified a protective locus for the *APOE4* allele in African-descent populations, which significantly lowers the risk for AD in *APOE4* carriers, in which the magnitude of the effect decreased from 7.2 to 2.1 for African carriers of the *APOE4* allele carrying this protective locus located 2MB from *APOE*. The protective haplotype has a frequency of 12% in African ancestry while being notably rare in Europeans, with a frequency of only 0.003%^18^.

Of note, *TRANK1*, a novel locus unraveled by a recent multi-ancestry meta-analysis in AD^8^, emerged as one of the top estimated PAR loci in the Black/African American ancestry in our PAR assessment. *TRANK1* seems to play an important role in African descent populations, as previously identified through a gene-based analysis in a GWAS conducted on African Americans^10^. Interestingly, this locus has also been highlighted in GWAS for schizophrenia and bipolar disorders, with the latter being well known to increase the risk of AD. Other important cross-ancestral AD loci that emerged in our study included *TSPAN14* for European and Black/African American ancestries, and *PICALM* in individuals of European, Latino, Black/African American, and East Asian descent. Both of these genes have been implicated in microglia activation, highlighting this pathway as a potential therapeutic target^18^.

In PD, our study identified *SNCA*, *MCCC1*, *MAPT*, and *VPS13C* as the loci displaying the highest PAR. Three of these loci are multi-signal, with different and significant impacts across multiple ancestries based on PAR estimates. In contrast, the rs10748818 variant at the *GBF1* locus was identified as the locus with the highest estimated PAR for the African/African Admixed population. This gene seems to modulate intracellular protein traffic, and variants in it have also been associated with PD in Chinese populations^19^. *HLA-DRB5* rs112485576 had the highest estimated PAR for the East Asian population. Variants in this locus have also been previously associated with ulcerative colitis and Crohn’s disease, suggesting a potential overlapping genetic nexus with gut disturbances in PD^20^. Of note, the PAR estimates for *GBA1* differ across populations, with the population-specific *GBA1* rs3115534 variant having one of the highest PAR estimates for the African/African Admixed ancestry, while other well-known coding *GBA1* variants showed varying PAR estimates depending on the population. On the other hand, the lower observed PAR estimates for the *LRRK2* p.G2019S variant across ancestries are expected since this variant is less common compared to the PD GWAS loci resulting in a smaller population attributable influence^9^. These results highlight the role of key cross-ancestral signals and suggest the potential of targeted therapies in these populations, emphasizing the importance of designing therapeutic approaches tailored to the unique genetic profiles of each ancestry.

This study prioritized potential genetic targets across diverse ancestries by estimating the PAR for genetic loci associated with AD and PD risk. Identifying genetic risk variants with consistent PARs across multiple ancestries highlights the promise of developing therapeutics with broader effectiveness, surpassing the constraints of population-specific treatments. Some limitations in this study include differences in sample sizes across populations assessed which may influence PAR estimates. Future refinement of PAR for the purposes of highlighting genetic targets can be achieved by utilizing subpopulation summary statistics. Further, genotyping arrays have been primarily Eurocentric leading to the potential of missed risk signals in underrepresented populations. PAR estimates may be improved with the use of whole genome sequencing data in future genome-wide association studies. Consideration of genetic and other risk factors, such as environmental, in the use of PAR could prove useful in future applications. Overall, our results indicate that concentrating on specific genetic loci could amplify the efficacy of targeted medical interventions, ultimately serving a wider population.

## Supplementary Material

Supplement 1
